# Effects of Oleanolic Acid Derived from Wine Pomace on Periodontopathic Bacterial Growth in Healthy Individuals: A Randomized Placebo-Controlled Study

**DOI:** 10.3390/dj12050133

**Published:** 2024-05-08

**Authors:** Kyoko Shimazu, Kouta Ookoshi, Satoshi Fukumitsu, Hiroyuki Kagami, Chieko Mitsuhata, Ryota Nomura, Kazuhiko Aida

**Affiliations:** 1 Innovation Center, Central Research Laboratory, Nippn Corporation, Yokohama 243-0041, Japan; k-ookoshi@nippn.co.jp (K.O.); sfukumitsu@nippn.co.jp (S.F.); kaida@nippn.co.jp (K.A.); 2 Kagami Dental Office, Kitahiroshima 061-1134, Japan; molom_nolon@yahoo.co.jp; 3 Department of Pediatric Dentistry, Graduate School of Biomedical and Health Sciences, Hiroshima University, Hiroshima 734-8553, Japan; chiekom@hiroshima-u.ac.jp (C.M.); rnomura@hiroshima-u.ac.jp (R.N.)

**Keywords:** oleanolic acid, periodontopathic bacteria, oral microbiome, periodontal disease, wine pomace

## Abstract

Periodontal disease is caused by oral pathogenic bacteria and is associated with systemic disease and frailty. Therefore, its prevention is crucial in extending healthy life expectancy. This study aimed to evaluate the effect of orally administered oleanolic acid, extracted from wine pomace, on periodontopathic bacterial growth in healthy individuals. In this randomized, placebo-controlled, double-blind, parallel-group comparison study, 84 healthy adults were assigned to a placebo (*n* = 29), low-dose (*n* = 29, 9 mg oleanolic acid), or high-dose (*n* = 26, 27 mg oleanolic acid) groups. The number of oral bacteria in their saliva, collected before and 5 h after administration, was determined using the polymerase chain reaction-invader technique. The proportion of periodontopathic bacteria among the total oral bacteria in the saliva was calculated. Oleanolic acid significantly decreased the proportion of *Porphyromonas gingivalis* among the total oral bacteria in a dose-dependent manner (*p* = 0.005 (low-dose) and *p* = 0.003 (high-dose) vs. placebo, Williams’ test). Moreover, high-dose oleanolic acid decreased the proportion of *Tannerella forsythia* (*p* = 0.064 vs. placebo, Williams’ test). Periodontopathic bacteria are closely associated with the development and progression of periodontal disease; thus, the continuous daily intake of oleanolic acid derived from pomace may be helpful in maintaining a healthy oral microbiome by controlling the proportion of periodontopathic bacteria.

## 1. Introduction

Dental caries and periodontal disease are among the most prevalent conditions worldwide [1,2]. These diseases are caused by oral bacteria. Periodontal disease begins with gingivitis, defined as inflammation caused by enzymes and toxins produced by oral bacteria localized to the gums; over time, it progresses to periodontitis, leading to the destruction of the periodontal ligament and alveolar bone when the inflammation becomes chronic. Periodontitis is irreversible and can lead to tooth loss [3]. As teeth are required for eating and communication, tooth loss results in physical health risks, including undernutrition and sarcopenia, as well as social frailty [4,5,6]. Furthermore, these bacteria are associated with various systemic diseases [7,8], including heart disease [9], respiratory infections [10], diabetes [11], tumors [12], brain disease [13], and risk of poor pregnancy outcomes [14]. Therefore, they significantly impact physical and mental health.

Multiple bacterial complexes are involved in periodontal tissue inflammation [15,16]. Among them, *Porphyromonas gingivalis* (*P. gingivalis*)*, Treponema denticola* (*T. denticola*)*,* and *Tannerella forsythia* (*T. forsythia*) are classified as red-complex high-risk periodontopathogens because they are associated with the onset and progression of severe periodontitis [17]. However, these bacteria are also involved in gingivitis and can be detected even in healthy people [18,19]. Furthermore, they are a keystone pathogen, despite their low abundance [20,21]. Therefore, controlling periodontopathic bacterial growth and maintaining a healthy oral microbiome are essential. Some chemical anti-agents, such as chlorhexidine, inhibit microbes; however, long-term treatment with these drugs generates resistant pathogens [22,23]. Therefore, new natural ingredients that are safe and effective are needed.

Red wine is rich in polyphenols, such as anthocyanins, resveratrol, and catechins, derived from grapes. Red wine and these polyphenols may have health benefits [24,25]. For example, they may help to prevent arteriosclerosis via the antioxidant effects [26]; interestingly, they could also help to prevent periodontal disease [27] through their anti-inflammatory [28] and antibacterial effects on related pathogens [29]. Annually, wine production yields 20 million tons of pomace, which is primarily processed as compost. Some of the useful components, such as oleanolic acid, remain in the pomace [30,31,32], because they are not transferred to the wine.

**Figure 1:**
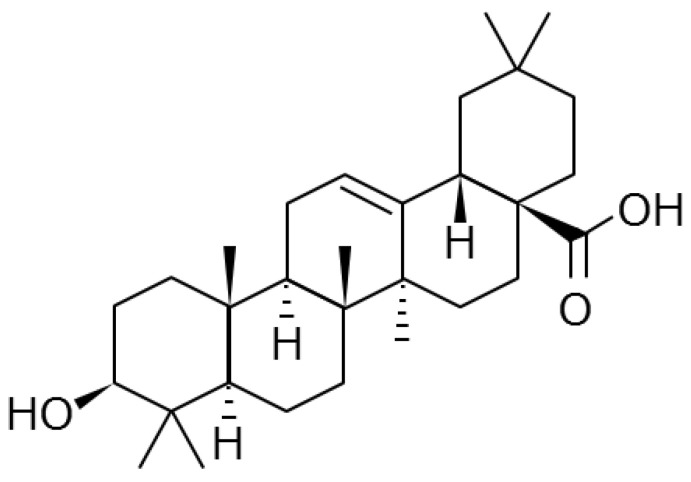
Structural formula of oleanolic acid.

Oleanolic acid is one of the most common pentacyclic triterpenes in nature (Figure 1). It is a constituent of cuticular wax, a lipid membrane that covers the epidermis of plants including grapes, apples, and olives, and it plays a role in protecting plants from external environmental stress [33]. Oleanolic acid also has antibacterial effects [34], as well as various physiological effects [35,36] including anti-inflammatory [37], antioxidant [38,39], antidiabetic [40], anticancer [41], neuroprotective [42], and hepatoprotective effects [43]. Triterpenes, such as oleanolic acid, derived from grapes or olives have been shown to suppress the growth of *P. gingivalis* in minimum inhibitory concentration (MIC) tests [44,45,46] and they prevented periodontitis by exerting anti-inflammatory effects in a rat model of acute periodontitis and in a mouse model of periodontitis [47,48]. The mechanism behind oleanolic acid’s anti-inflammatory effects is the blockage of signal transduction pathways; this involves inhibiting the phosphorylation of nuclear factor-kappa B (NF-kB) and signal transducers and activators of transcription 1 (STAT 1), thereby suppressing the expression of inflammatory cytokines [36]. Notably, oleanolic acid has been reported to directly bind to the glucosyltransferase produced by *Streptococcus mutans* (*S. mutans*) and subsequently inhibit its enzymatic activity, thereby suppressing plaque formation [49,50]. However, no reports have verified the effect of oleanolic acid on periodontal disease in humans. Oleanolic acid extracted from wine pomace effectively inhibits the growth of *S. mutans* and *P. gingivalis* in MIC tests [51]. Regarding dental caries, the intake of this extract (36 mg of oleanolic acid as the active ingredient per day for 4 days) suppressed the growth of *S. mutans* in the oral cavity in a clinical study [52]. This study hypothesized that orally administered oleanolic acid extracted from pomace would also control periodontopathic bacterial growth in the human oral cavity, which has been observed in in vitro and animal tests [44,45,46]. Therefore, this study aimed to examine the effect of oleanolic acid tablets, containing 27 mg or 9 mg of oleanolic acid, on periodontopathic bacterial growth in human saliva. This research was conducted as a first step toward creating a safe and easy-to-use oral care food material that can prevent dental caries and periodontal disease.

## 2. Materials and Methods

### 2.1. Participants and Study Design

This study was conducted in accordance with the Declaration of Helsinki of 1975, as revised in 2013, the Ethical Guidelines for Medical and Health Research Involving Human Subjects, and the Act on the Protection of Personal Information. The study was approved by the Institutional Review Board of the Social Medical Corporation, Sokujinkai Kitahiroshima Hospital (Kitahiroshima, Japan; protocol number: 008; date of approval: 31 October 2022) and was registered as a clinical trial with the University Hospital Medical Information Network–Clinical Trials Registry (UMIN–CTR; trial registration numbers: UMIN000049515 and UMIN000049528). This trial was conducted from November 2022 to December 2022 in Japan. All participants provided informed consent before participating in the study.

**Figure 2:**
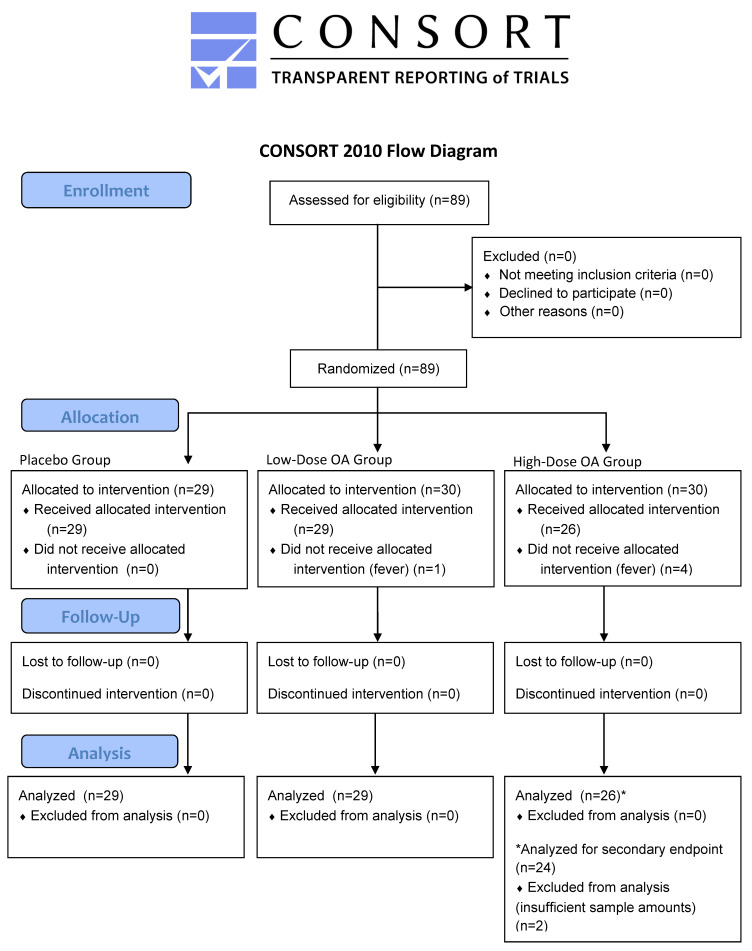
Flow diagram of the study. CONSORT—Consolidated Standards of Reporting Trials. OA—oleanolic acid.

The recruited individuals were healthy adults, aged 20–60 years, who were not receiving treatment for any disease, including oral diseases, and were not receiving regular dental maintenance. Additionally, they had not taken antibiotics or antibacterial drugs within 1 month before the commencement of this study, were nonsmokers, and were not pregnant. They also did not consume any medications, health foods, or supplements that may have influenced the results of this study. Furthermore, they did not habitually consume excessive amounts of grapes or grape-related products, including juice, raisins, or wine, daily. They were recruited broadly from the general public rather than from any specific population. It was confirmed that the participants met the criteria through a self-report survey. This study utilized two different test tablets, each with varying levels of oleanolic acid, along with a placebo tablet devoid of this extract. In this randomized, placebo-controlled, double-blind, parallel-group comparative study, a total of 89 healthy adults were allocated to three groups: the placebo group (*n* = 29, no oleanolic acid), the low-dose OA group (*n* = 30, 9 mg of oleanolic acid), and the high-dose OA group (*n* = 30, 27 mg of oleanolic acid) (Figure 2). The allocation was performed using the stratified randomization method, utilizing the number of *P. gingivalis* bacteria identified in a preliminary oral bacteria test as the stratification factor. The allocation process was conducted by an individual not directly involved in the study, using computer-generated random numbers. The participants were evenly distributed into the placebo group, low-dose OA group, and high-dose OA group in a 1:1:1 ratio. The allocator strictly managed the allocation details until the study director provided instructions for unblinding. Regarding blinding, this study was conducted without revealing the group allocation to the investigators, participants, and assessors; specifically, arbitrary numbers were assigned to the groups by the allocator, and only they were aware of these details. Additionally, the test tablets were indistinguishable as to whether they contained the active ingredient. The participants, researchers, and assessors were informed by the allocator of the details, i.e., which number corresponded to which group, after the analysis was fully completed. The sample size was determined based on methods described in previous studies [53,54] examining the effects of oral bacteria in human saliva. After excluding five participants who could not participate due to fever, this study included a final sample of 84 participants (45 men and 39 women; mean age, 45.1 years), divided into the following groups: placebo group—13 men and 16 women (*n* = 29), mean age, 45.0 ± 8.1 years; low-dose OA group—17 men and 12 women (*n* = 29), mean age, 45.3 ± 10.6 years; and high-dose OA group—15 men and 11 women (*n* = 26), mean age, 45.1 ± 10.2 years.

### 2.2. Method of Intake

The participants licked and dissolved the assigned tablet in their mouths, rather than chewing or swallowing them whole. Their saliva was collected before and 5 h after administration. The test period spanned one day. During this period, they were required to fast (only water was allowed, with no drinking permitted within 2 h of saliva collection). Additionally, they were instructed to abstain from all oral hygiene practices, including brushing, flossing, and mouth rinsing, from the moment that they woke up until the completion of the test on that day. They were also prohibited from drinking alcohol, including wine, on the day before the test.

### 2.3. Test Foods

Oleanolic acid was extracted from red wine pomace using ethanol and then purified. Subsequently, the extract was formulated to contain 9 mg or 27 mg of oleanolic acid as the active ingredient in one tablet (diameter: 16 mm, weight: 1000 mg per tablet), and they were then compressed with dextrin, cellulose, silicon dioxide, and calcium stearate for the tableting process. The placebo tablets were identical in appearance but contained dextrin in place of the extract. These tablets were manufactured by Shefco Co., Ltd. (Tokyo, Japan).

### 2.4. Evaluation Methods

#### 2.4.1. Efficacy Evaluation

Saliva samples were obtained from the participants, and the counts of periodontopathic bacteria and total oral bacteria were assessed using the polymerase chain reaction (PCR) invader technique [55]. The ratio of each periodontopathic bacteria to the overall oral bacteria was subsequently calculated. The rate of change before and after the consumption of the test product was calculated for each participant. Next, this average value was assessed to determine intergroup differences.

##### Saliva Collection

A complete saliva sample was collected after each participant chewed paraffin gum for 5 min, before and 5 h after taking the tablet. Next, the collected saliva (0.5 mL) was transferred into a tube using a dropper and utilized as the sample.

##### Determining the Number of Bacteria

The deoxyribonucleic acid (DNA) present in each sample was extracted using a silica column and commercial kit (DNA blood mini kit, QIAGEN K.K., Tokyo, Japan). The counts of the periodontopathic bacteria and total oral bacteria in the saliva were determined using the PCR invader technique [55]. Subsequently, the proportion of periodontopathic bacteria relative to the total oral bacteria, based on the obtained measurements, was calculated. The BML Co., Ltd. (Tokyo, Japan) measured the bacterial counts.

#### 2.4.2. Safety Evaluation

The safety assessment involved the examination of adverse events and concurrent symptoms, which were evaluated by confirming a range of subjective and objective indicators through interviews and questionnaires.

### 2.5. Statistical Analysis

All measured values are expressed as the mean value ± the standard deviation. The differences among the three groups regarding the proportion of periodontopathic bacteria among the total number of oral bacteria before administration were evaluated using a one-way analysis of variance. The differences among the three groups regarding the rate of change in the proportion of periodontopathic bacteria before and after administration were evaluated using Williams’ test [56]; this was because the effect of oleanolic acid on periodontopathic bacterial growth was presumed to be dose-responsive, based on the findings of in vitro studies [49,57]. All statistical analyses were performed using the R version 4.0.3 software (Institute for Statistics and Mathematics, Vienna, Austria; www.r-project.org, accessed on 25 April 2023). Statistical significance was set at *p* < 0.05.

## 3. Results

### 3.1. Effect on Periodontopathic Bacteria in the Oral Cavity

All 84 participants (45 men and 39 women; mean age: 45.1 years) completed the trial. We were able to quantify the counts of periodontopathic bacteria, specifically *P. gingivalis*, *T. denticola*, and *T. forsythia*, as well as the total oral bacteria, in the saliva collected before and 5 h after the ingestion of a placebo or test tablet (i.e., low-dose or high-dose). This measurement was accomplished using the PCR invader method [55], followed by the calculation of the ratio of periodontopathic bacteria to total oral bacteria. Additionally, we were able to compute the rate of change in the proportion of the target periodontopathic bacteria before and after tablet ingestion for each participant and compared the average values across the groups. The primary endpoint was the rate of change in the proportion of *P. gingivalis*, while the secondary endpoint was the rate of change in the proportion of *T. denticola* or *T. forsythia*. The secondary endpoint analysis in the high-dose OA group was performed on a reduced sample size of *n* = 24 due to insufficient sample amounts for measurement. Notably, there were no significant differences among the three groups in the percentage of periodontopathic bacteria before tablet administration.

#### 3.1.1. Changes in the Proportion of *P. gingivalis*

**Figure 3:**
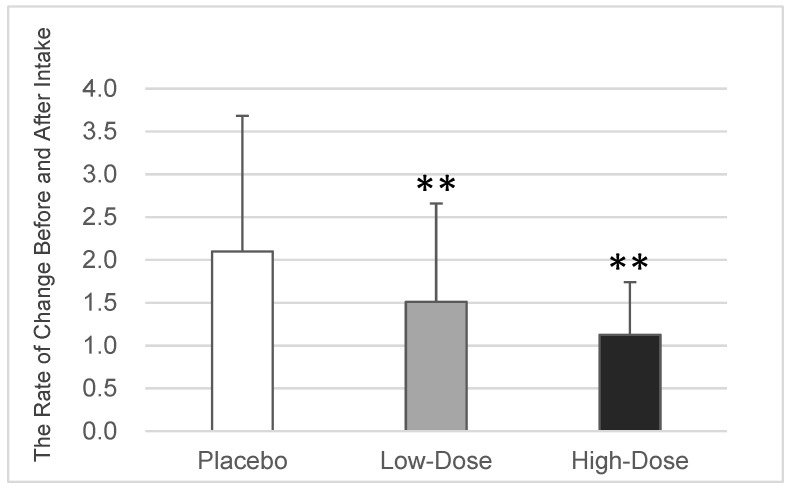
Changes in the proportion of *P. gingivalis*. The average value ± the standard deviation of the rate of change in the proportion of *P. gingivalis* among the total oral bacteria before and after the intake of the placebo or oleanolic acid for each participant is displayed (*n* = 29/29/26 for the placebo, low-dose OA, and high-dose OA groups, respectively) ** *p* < 0.01 vs. the placebo group, based on Williams’ test. OA—oleanolic acid.

In the placebo group (*n* = 29), the average rate of change in the proportion of *P. gingivalis* among the total oral bacteria was 2.10 ± 1.58. In comparison, the low-dose OA group (*n* = 29) had a rate of change of 1.51 ± 1.15 (*p* = 0.005 vs. the placebo group, based on Williams’ test), while the high-dose OA group (*n* = 26) had a rate of change of 1.13 ± 0.61 (*p* = 0.003 vs. the placebo group, based on Williams’ test). These findings indicated a significant and dose-dependent reduction in the growth of the proportion of *P. gingivalis* among the total oral bacteria with the consumption of oleanolic acid (Table 1 and Figure 3).

#### 3.1.2. Changes in the Proportion of *T. denticola*

**Figure 4:**
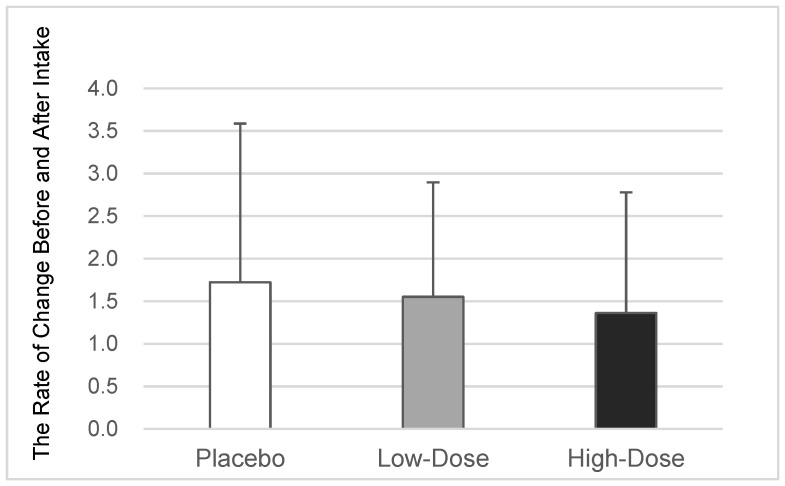
Changes in the proportion of *T. denticola*. The average value ± the standard deviation of the rate of change in the proportion of *T. denticola* among the total oral bacteria before and after the intake of the placebo or oleanolic acid for each participant is displayed (*n* = 29/29/24 for the placebo, low-dose OA, and high-dose OA groups, respectively) vs. the placebo group, based on Williams’ test. OA—oleanolic acid.

In the placebo group (*n* = 29), the mean rate of change in the proportion of *T. denticola* among the total oral bacteria was 1.72 ± 1.86. In comparison, the low-dose OA group (*n* = 29) had a rate of change of 1.55 ± 1.34 (*p* = 0.310 vs. the placebo group, based on Williams’ test), while the high-dose OA group (*n* = 24) had a rate of change of 1.36 ± 1.42 (*p* = 0.267 vs. the placebo group, based on Williams’ test). These results indicated no significant differences in the proportion of *T. denticola* among the three groups, although the intake of oleanolic acid appeared to have a tendency to reduce the growth in the proportion of *T. denticola* among the total oral bacteria in a dose-dependent manner (Table 1 and Figure 4).

#### 3.1.3. Changes in the Proportion of *T. forsythia*

**Figure 5:**
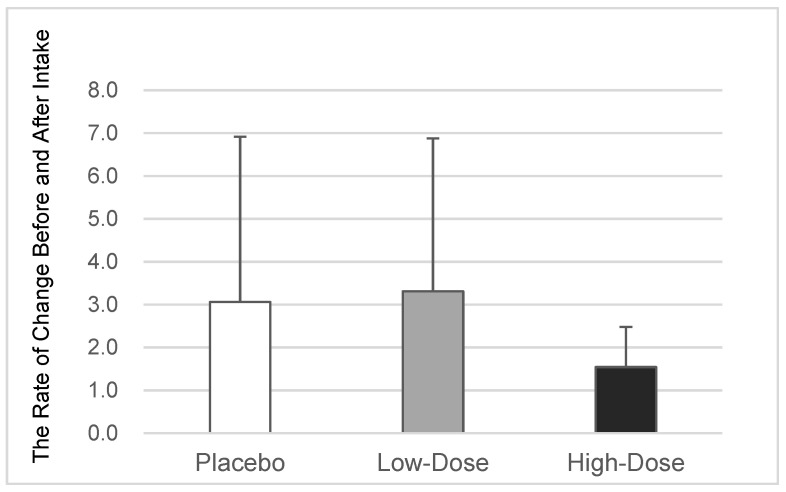
Changes in the proportion of *T. forsythia*. The average value ± the standard deviation of the rate of change in the proportion of *T. forsythia* among the total oral bacteria before and after the intake of the placebo or oleanolic acid for each participant is displayed (*n* = 29/29/24 for the placebo, low-dose OA, and high-dose OA groups, respectively) vs. the placebo group, based on Williams’ test. OA—oleanolic acid.

In the placebo group (*n* = 29), the mean rate of change in the ratio of *T. forsythia* to the total number of oral bacteria was 3.06 ± 3.86. In contrast, the low-dose OA group (*n* = 29) had a rate of change of 3.31 ± 3.57 (*p* = 0.296 vs. the placebo group, based on the Williams test), and the high-dose OA group (*n* = 24) had a rate of change of 1.54 ± 0.94 (*p* = 0.064 vs. the placebo group, based on Williams’ test). A noteworthy finding is that high-dose oleanolic acid intake tended to reduce the proportion of *T. forsythia* among the total oral bacteria (*p* = 0.064, as indicated in Table 1 and Figure 5), although there was no significant difference between the low-dose OA group and the other two groups.

### 3.2. Occurrence of Adverse Events

Various subjective and objective symptoms were confirmed through interviews and questionnaires. No adverse events caused by the test tablet’s administration were noted in any of the participants.

## 4. Discussion

This study aimed to evaluate the effect of oleanolic acid extracted from wine lees (i.e., pomace) on the growth of periodontopathic bacteria, seeking to prevent periodontal disease in humans. Overall, 84 healthy adults (45 men and 39 women; mean age: 45.1 years) were assigned to the placebo group (*n* = 29, no oleanolic acid), low-dose OA group (*n* = 29, 9 mg of oleanolic acid), or high-dose OA group (*n* = 26, 27 mg of oleanolic acid). The participants received tablets based on their respective groups. The proportion of periodontopathic bacteria among the total oral bacteria was determined using the PCR invader technique [55] on saliva samples, which were collected before and after administration. Saliva or subgingival plaque can be collected to assess the number of periodontopathic bacteria. It has been confirmed that both sample types are valid [58] and have a significant positive correlation [59,60]. Therefore, in this case, saliva collection was chosen because it was easy to collect a large number of samples with this method, as the study aimed to evaluate the changes before and after the intake of the test tablet. Moreover, the PCR invader technique has detection sensitivity and reproducibility equivalent to or better than those of conventional real-time PCR [55,61]; it has been established and widely used as a method to evaluate the number of periodontopathic bacteria [62,63,64]. The average values of the rate of change before and after the intake of the test food were evaluated for each participant across the groups in this test. Accurately determining whether a test food enhances or inhibits a target bacterium when assessing the average values after test food administration in each group can be challenging [52]. This challenge arises due to the significant variations among individuals in the number or percentage of total oral bacteria, as well as the presence of specific bacteria, including cariogenic and periodontopathic strains. This study was no exception; it was found that the proportion of each periodontopathic bacterium among the total bacterial population in the saliva exhibited a wide range of variation. For example, the proportion of *P. gingivalis* ranged from 0.00011% to 0.29% (Table 1). Consequently, assessing the impact of the test food on the growth of the target bacteria proved challenging. Thus, the average rate of change before and after the intake of the test food for each participant in this study was evaluated. The results revealed that the oleanolic acid derived from pomace significantly decreased the proportion of *P. gingivalis* among the total oral bacteria in a dose-dependent manner (Figure 3, *p* = 0.005 (9 mg) and *p* = 0.003 (27 mg) vs. the placebo group, based on Williams’ test). Moreover, high-dose oleanolic acid tended to decrease the proportion of *T. forsythia* among the total oral bacteria (Figure 5, *p* = 0.064 vs. the placebo group, based on Williams’ test).

Periodontal pathogens classified within the red complex are also detected in healthy oral conditions and in the early stages of gingivitis, although these bacteria have been reported to be associated with severe periodontitis. Furthermore, reports have indicated that these bacteria can affect the oral flora, even at low doses, leading to the onset or worsening of periodontal disease [20,21]. Therefore, controlling the population of periodontopathic bacteria involved in inflammation is an effective approach to mitigating the onset of periodontal disease [65,66,67]. It has also been reported that red wine and polyphenols may have antimicrobial activity against periodontal pathogens [28,29]. However, this study focused on oleanolic acid, which does not transfer to wine, from the perspective of the Sustainable Development Goals. Oleanolic acid has been reported to inhibit the growth of periodontopathic bacteria, including *P. gingivalis*, *Actinobacillus actinomycetemcomitans*, and *Fusobacterium nucleatum*, as demonstrated in MIC tests [44,45,46]. More than 700 types of bacteria inhabiting the oral cavity have been reported [68]. This study’s findings emphasize the potential efficacy of oleanolic acid derived from pomace in inhibiting the growth of *P. gingivalis* and *T. forsythia*, both of which belong to the red complex, within the human oral environment.

Oleanolic acid has been reported to have a specific antibacterial effect on oral streptococci such as *S. mutans* and *Streptococcus sobrinus* [49,69], which cause dental caries, in addition to periodontopathic bacteria [44,45,46]. The mechanism of oleanolic acid’s antibacterial effect impacts the biosynthesis of peptidoglycan, a constituent of the cell envelope, thereby resulting in changes in cell morphology [70,71,72]. Additionally, oleanolic acid is associated with various physiological functions [73], including the oxidative stress response, the cell membrane, transport, transferases, energy biosynthesis, cell morphology, and the expression of genes related to ribosomes [73]. However, the range of its antibacterial spectrum and the mechanism of specificity have not yet been fully elucidated. Kozai et al. [49] reported that oleanolic acid acts as a bacteriostatic and bactericidal agent at low and high concentrations, respectively. Consistent with these findings, the oleanolic acid derived from pomace suppressed the increase in the proportions of *P. gingivalis* and *T. forsythia*, rather than reducing their numbers, in this study. Thus, oleanolic acid derived from pomace may effectively control bacteria and maintain good oral flora without causing bacterial replacement or the appearance of resistant bacteria.

This study had some limitations, such as the relatively small sample size, short test period, limited participant age range, limited study population with healthy oral conditions, and limited evaluation parameters. The total number of bacteria in the oral cavity is constantly changing; thus, it varies greatly depending on the day and collection time in individuals, and it is greatly influenced by their lifestyle habits, including oral hygiene and diet. Therefore, the test period was set to one day in this study, and the participants restricted their oral hygiene behaviors and diet during the test, as well as alcohol consumption on the day before the test. The inclusion and exclusion criteria were also set to exclude participants with factors that could influence the test results. The factors included diet (grapes and grape-related products such as raisins and wine), supplements for oral care, smoking habits, and medical history, including diabetes; this approach aimed to eliminate these external factors as much as possible and enable the evaluation of oleanolic acid’s effects on the oral bacteria. However, the possibility that other confounding factors were involved in the results cannot be completely excluded. A previous study [52], assessing the growth of *S. mutans* in humans, demonstrated that the continuous consumption of oleanolic acid extracted from pomace at a daily dosage of 27–36 mg for 4 days suppressed bacterial growth in the saliva in treated individuals compared with the placebo group. Furthermore, in the preliminary study, the continuous intake of this extract for 4 days reduced the presence of *P. gingivalis* in the saliva without affecting the total number of oral bacteria. Therefore, the growth-inhibiting effect on *P. gingivalis* observed in this study is expected to be maintained with continued intake, although only the proportion of periodontopathic bacteria in the saliva within 1 day was evaluated as a test period in this study.

Generally, older adults frequently exhibit advanced periodontal disease [74,75]. Furthermore, the prevalence of these periodontopathic bacteria in children is lower than that in adults, although their presence has been reported [76,77]. Additionally, scientists have noted that the infection is not constant and varies between the infected and non-infected states depending on the timing of the measurements. The participants in this study were presumed to have a relatively good state of oral health, without severe gingivitis or periodontitis, because individuals undergoing dental treatment or maintenance were excluded. However, the effect of oleanolic acid on the growth of periodontopathic bacteria would be expected to have the same effect in the target age group in this study and for a wide range of other age groups (from children to elderly individuals) because the functional mechanism of oleanolic acid should not be affected by age. Furthermore, it may be possible to verify the effectiveness of oleanolic acid in treating periodontal disease by evaluating its impact on the bacterial count and periodontal disease symptoms in not only healthy people but also patients with periodontal health issues. However, this factor remains unclear without further studies targeting a wider age range or other oral health conditions.

Oleanolic acid is widely known to exert anti-inflammatory effects [36] and suppress plaque formation [49,50]. Plaque is a pathogenic-determining factor because it favors the survival of bacteria within the host, thereby leading to the development of dental caries and periodontitis [78]. Therefore, the long-term intake of oleanolic acid may be effective in promoting healthy oral flora and preventing periodontal disease. This is attributed to its specific antibacterial properties against cariogenic and periodontopathic bacteria, its anti-inflammatory effect, and its inhibitory effect on plaque formation. However, the gingival index, bleeding on probing, probing depth, and plaque index following long-term intake should be evaluated. Overall, the continuous daily intake of oleanolic acid derived from pomace, may be helpful in maintaining a healthy oral microbiome by controlling the proportion of periodontopathic bacteria. Further research could lead to the creation of a safe and easy-to-use oral care material that can be used to prevent or treat periodontal disease.

## 5. Conclusions

This study investigated the proportion of periodontopathic bacteria in the saliva of healthy adults aged 20–60 years after the intake of oleanolic acid (9 mg or 27 mg) as an active ingredient. The intake of oleanolic acid in one day reduced the proportion of periodontopathic bacteria. Periodontopathic bacteria are closely associated with the development and progression of periodontal disease; thus, the findings of this study suggest that the continuous intake of oleanolic acid derived from wine pomace may help maintain a healthy oral microbiome.

## Figures and Tables

**Table 1:** The proportion of periodontopathic bacteria among the total oral bacteria in the saliva.

	Placebo Group (*n* = 29/29/29)			Low-Dose OA Group (*n* = 29/29/29)				High-Dose OA Group (*n* = 26/24/24)			
	Before (%)	After (%)	Rate of Change	Before (%)	After (%)	Rate of Change	*p* Value	Before (%)	After (%)	Rate of Change	*p* Value
Proportion of *P. gingivalis* (0.00011%–0.29%)	0.031 ± 0.033	0.041 ± 0.035	2.10 ± 1.58	0.043 ± 0.048	0.054 ± 0.062	1.51 ± 1.15	0.005 **	0.061 ± 0.072	0.048 ± 0.043	1.13 ± 0.61	0.003 **
Proportion of *T. denticola* (0.000077%–0.032%)	0.0033 ± 0.0036	0.0048 ± 0.0057	1.72 ± 1.86	0.0032 ± 0.0040	0.0034 ± 0.0042	1.55 ± 1.34	0.310	0.0047 ± 0.0070	0.0042 ± 0.0059	1.36 ± 1.42	0.267
Proportion of *T. forsythia* (0.0014%–0.36%)	0.030 ± 0.029	0.055 ± 0.069	3.06 ± 3.86	0.030 ± 0.049	0.045 ± 0.041	3.31 ± 3.57	0.296	0.048 ± 0.062	0.053 ± 0.048	1.54 ± 0.94	0.064

The average value ± the standard deviation of the proportion of each periodontopathic bacterium among the total oral bacteria before and after the intake of the placebo or oleanolic acid. The term “rate of change” refers to the average value ± the standard deviation of the rate of change before and after the intake of the placebo or oleanolic acid for each participant. The sample sizes for each group are shown (*n* = proportion of *P. gingivalis*/proportion of *T. denticola*/proportion of *T. forsythia*). The values in parentheses indicate the minimum to maximum percentages of periodontopathic bacteria. ** *p* < 0.01 vs. the placebo group, based on Williams’ test. OA—oleanolic acid.

## Data Availability

The data that support the findings are available from the corresponding author with the permission of the Institutional Review Board of the Social Medical Corporation Sokujinkai Kitahiroshima Hospital (Kitahiroshima, Japan).
